# Myosin 1b is an actin depolymerase

**DOI:** 10.1038/s41467-019-13160-y

**Published:** 2019-11-15

**Authors:** Julien Pernier, Remy Kusters, Hugo Bousquet, Thibaut Lagny, Antoine Morchain, Jean-François Joanny, Patricia Bassereau, Evelyne Coudrier

**Affiliations:** 1 Laboratoire Physico-Chimie Curie, Institut Curie, PSL Research University, CNRS UMR168, 75005 Paris, France; 2 Sorbonne Université, 75005 Paris, France; 3 Institut Curie, PSL Research University and C.N.R.S. UMR 144, 26 rue d’Ulm, Paris, France; 4 University Paris Descartes, Center for Research and Interdisciplinarity (CRI), 8 Rue Charles V, Paris, France; 5 ESPCI Paris, PSL Research University, 10 rue Vauquelin, 75005 Paris, France; 6 Collège de France, 11 Place Marcelin Berthelot, 75231 Paris Cedex05, France

**Keywords:** Motor protein function, Cell biology

## Abstract

The regulation of actin dynamics is essential for various cellular processes. Former evidence suggests a correlation between the function of non-conventional myosin motors and actin dynamics. Here we investigate the contribution of myosin 1b to actin dynamics using sliding motility assays. We observe that sliding on myosin 1b immobilized or bound to a fluid bilayer enhances actin depolymerization at the barbed end, while sliding on myosin II, although 5 times faster, has no effect. This work reveals a non-conventional myosin motor as another type of depolymerase and points to its singular interactions with the actin barbed end.

## Abstract (web summary)

Former evidence suggests a correlation between the function of non-conventional myosin motors and actin dynamics. Here authors use in vitro assays in which they observe that actin sliding on myosin 1b immobilized or bound to a fluid bilayer enhances actin depolymerization at the barbed end.

## Introduction

Actin filaments (F-actin) form a variety of dynamical architectures that govern cell morphology and cell movements. The dynamics of the actin networks are regulated in space and time by the assembly and disassembly of actin polymers under the control of regulatory proteins. Cortical actin organizes lateral movement of transmembrane proteins and participates in membrane signaling by interacting transiently with the plasma membrane^1^. One class of actin-associated molecular motors, the single-headed myosin 1 proteins, bridges cortical actin to the plasma membrane. Polymerization of actin filaments at the plasma membrane generates forces on the membrane as well as on their membrane linkers. Inversely myosin 1 can exert and sustain pN forces on F-actin^2^.

**Fig. 1:**
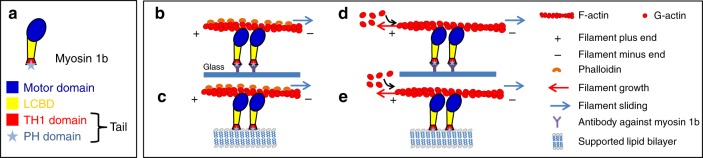
Myo1b-Actin gliding assays. **a** Schematic representation of domain organization of Myo1b. Motor domain (blue); Light Chain Binding Domain (LCBD) (yellow); TH1 domain (red), PH domain (cyan) that binds phosphoinositides. **b**–**e** Gliding assays of stabilized actin filaments (**b**–**c**) and polymerizing actin filaments (**d**–**e**) sliding on Myo1b anchored on coverslip (**b**–**d**) or bound to a supported lipid bilayer (**c**–**e**)

This important class of myosins contains a motor domain at its N-terminus that binds F-actin in response to ATP hydrolysis, a light chain binding domain (LCBD) that binds calmodulin (in most cases), and a Tail domain at the C-terminus (Fig. 1a)^3^. The Tail domain encompasses a tail homology domain (TH1) with a pleckstrin homology motif (PH) that binds phosphoinositides (Fig. 1a). Beside the involvement of myosin 1 proteins in a large variety of cellular processes including cell migration and membrane trafficking^3^, manipulation of myosin 1 expression has revealed a correlation between these myosins and actin network architecture^4–7^. In particular, under- or overexpression of one of these myosins, myosin 1b (Myo1b), affects the organization of the actin cytoskeleton in the juxtanuclear region of HeLa cells^4^ and in growth cones of cortical neurons^6^. In contrast to muscle Myosin II (MyoII), this particular Myo1b is a catch-bound motor (the time Myo1b remains bound to F-actin strongly increases with an applied load), it thus remains attached to the filament for a time that depends on the applied force^8^. Due to its mechanosensitive behavior, Myo1b could in turn exert a force on actin filaments^8,9^ and thus affect their polymerization. However, the role of these motors in actin dynamics remains to be explored. In this paper, we use in vitro F-actin gliding assays (Fig. 1b–e) and total internal reflection fluorescence (TIRF) microscopy to study the effect of full-length Myo1b on actin polymerization dynamics, with the motors either immobilized on a solid substrate (Fig. 1b, d) or bound to a fluid supported bilayer, which mimics cell membranes (Fig. 1c, e).

## Results

### F-actin depolymerizes when sliding on immobilized Myo1b

**Fig. 2:**
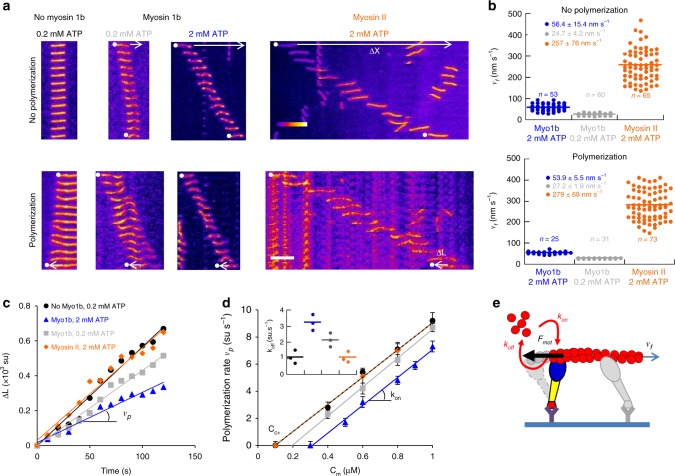
Sliding on immobilized myosin 1b increases F-actin depolymerization. **a** Representative kymographs of stabilized F-actin (top) or polymerizing F-actin with 0.6 µM G-actin (bottom), on uncoated glass or sliding on glass coated with Myo1b (2 mM and 0.2 mM ATP (see Supplementary Movies 2 and 3) or MyoII (see Supplementary Movie 6). The sliding distance *ΔX* and the elongation *ΔL* of the filaments are indicated by white arrows. Actin fluorescence intensity is represented according to the Fire LUT of Image J. Scale bar, 5μm. 1 image/10 s. **b** Dot plot representation of the sliding velocities *v*_*f*_ of stabilized (top) and polymerizing actin filaments (0.6 µM G-actin) (bottom) on immobilized Myo1b (8000 molecules/μm^2^) at 2 mM (blue) or 0.2 mM (gray) ATP or sliding on MyoII at 2 mM ATP (orange). The number of analyzed filaments and the mean-values ± s.e.m. are indicated. **c** Filament elongation Δ*L* (normalized by the length of the actin subunit (su) equal to 2.7 nm) versus time for filaments shown in A (bottom) in the absence of myosins and in the presence of MyoII or Myo1b at two ATP concentrations. The polymerization rate at the barbed end *v*_*p*_ (in su/s) is deduced from the slope. **d** *v*_*p*_ as a function of G-actin concentration *C*_*m*_ for the different conditions. The fits correspond to $v_p = k_{on}C_m - k_{off}$, with *k*_*on*_ the rate of association of G-actin and *k*_*off*_ the rate of dissociation. $C_{c + }$ is the critical concentration for polymerization. Inset: *k*_*off*_ for the different conditions. Error bars represent s.e.m. (*n* > 25). Source data are provided as a Source Data file. **e** Model for the role of Myo1b motor on the dissociation (depolymerization) rate *k*_*off*_. The filament, sliding at velocity *v*_*f*_, experiences a force *F*_mot_ at the barbed end while the motor is attached, thus impacting *k*_*off*_, but not the association (polymerization) rate *k*_on_

We first measured the sliding velocity *v*_*f*_ of single stabilized F-actin on Myo1b immobilized on a glass coverslip (Supplementary Fig. 1a, top and Supplementary Movie 1), the sliding velocity *v*_*f*_ and the polymerization rate *v*_*p*_ (expressed in actin sub-unit/s, with the length of an actin subunit being equal to 2.7 nm) of single F-actin (Supplementary Fig. 1a, bottom and Supplementary Movie 1) (“Methods”), both in the presence and in the absence of 0.3% methylcellulose for keeping the filaments in the TIRF field, by image analysis. At high Myo1b density (8000 µm^−2^) (for the motor density measurement, see the “Methods” section and Supplementary Fig. 1b), both stabilized and polymerizing filaments move with the same average sliding velocity *v*_*f*_  = 56.4 ± 15.4 nm s^−1^ and *v*_*f*_ = 53.9 ± 5.5 nm s^−1^, respectively (Fig. 2a, b, Supplementary Movie 1 and Supplementary Table 1) in the presence of 2 mM ATP (above saturation for motor activity)^10^. In both cases, this velocity decreases by about a factor two when decreasing the Myo1b density by a factor of twenty (Supplementary Fig. 2b, c, Supplementary Table 1) or when reducing the ATP level to 0.2 mM (Fig. 2a, b, Supplementary Movies 2 and 3) below saturation for Myo1b, but not affecting actin polymerization (Supplementary Table 2).

We next investigated the impact of Myo1b on actin polymerization upon filament sliding. The actin assembly-disassembly kinetics are an order of magnitude faster at the barbed (plus) end than at the pointed (minus) end^11^. Thus, we measured the elongation Δ*L* of F-actin at the barbed-end versus time (Fig. 2c). Strikingly, filament sliding on Myo1b decreases the actin polymerization rate *v*_*p*_, as compared to actin polymerization in the absence of Myo1b (Fig. 2d and Supplementary Movie 3). This effect is stronger for high filament sliding velocity (in the presence of 2 mM ATP) and weaker at lower Myo1b density on the substrate (Supplementary Fig. 2b, d, Supplementary Movie 3 and Supplementary Table 2). We also measured the dynamics of the pointed (minus) end by detecting the relative movement of this extremity compared to a fiducial point on the filament. In contrast with the barbed end, we did not observe any filament length variation (Supplementary Fig. 2a and Supplementary Movie 4), thus filament sliding on the motors reduces the actin polymerization rate at the barbed-end only. As a control, we tested the impact on actin polymerization of free Myo1b present only in the bulk, or immobilized on the surface but inactivated (Supplementary Fig. 2b, d and Supplementary Movie 5); we did not observe any effect on polymerization (Supplementary Fig. 2e). Moreover, although actin filaments slide five-fold faster on non- or weak catch-bond myosins such as muscle myosin II (MyoII)^12^, at the same bulk monomeric-actin (G-actin) concentration (Fig. 2a, b and Supplementary Movie 6), the actin polymerization rate remains similar to the control (Fig. 2c, d). These observations demonstrate that an immobilized Myo1b motor with intact activity reduces the actin polymerization rate at the barbed-end up to a factor two (Fig. 2d and Supplementary Table 2) in contrast to muscle MyoII. One important characteristic of Myo1b compared to MyoII that could be relevant, is that it is a catch-bond motor.

Dynamics at the barbed-end results from a balance between the rate of association of G-actin *k*_on_ and the rate of dissociation *k*_off_ (Fig. 2e); steady state is obtained at the critical concentration $C_{c^ + }$. Classically, these dynamical parameters are deduced from the measurement of the variation of the polymerization rate *v*_*p*_with G-actin concentration *C*_*m*_: $v_p = k_{\mathrm{on}}C_m - k_{\mathrm{off}}$. By varying the G-actin bulk concentration from 0.1 to 1 μM in the presence of either 0.2 mM and 2 mM ATP, we observed that the slope corresponding to *k*_on_ is unchanged when F-actin slides over Myo1b, whereas $C_{c^ + }$ which is the ratio between *k*_off_ and *k*_on_ increases (Fig. 2d) demonstrating that *k*_off_ increases under these conditions (Fig. 2d and Supplementary Table 2). Still, in the absence of G-actin in the bulk, filaments depolymerize faster when they slide over Myo1b (Supplementary Fig. 2f, g and Supplementary Movie 7). Interestingly, the dissociation rate is weakly affected when reducing Myo1b density, similarly to sliding velocity (Supplementary Fig. 2e and Supplementary Table 2). In contrast, while sliding on MyoII is much faster, this myosin has no influence on *k*_off_ at the barbed-end of the filament (Fig. 2d and Supplementary Table 2). Together, these observations indicate that the Myo1b is an actin depolymerase.

### F-actin depolymerizes when sliding on Myo1b bound to SLB

**Fig. 3:**
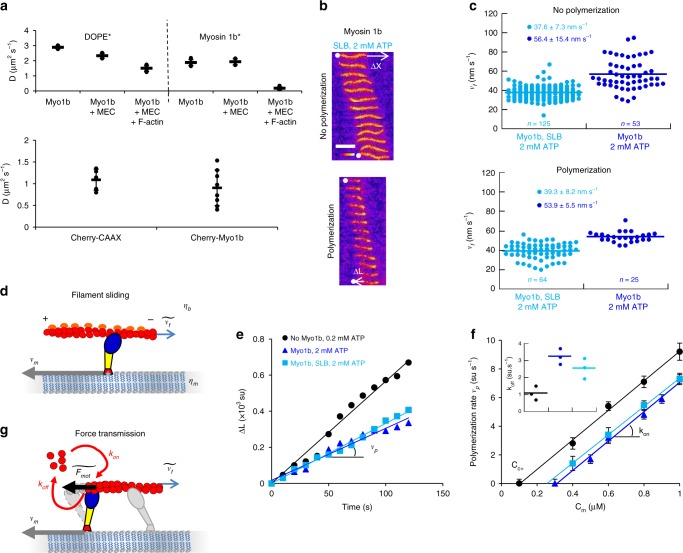
Sliding on myosin 1b bound to SLB increases F-actin depolymerization. **a** Top: Diffusion coefficients of Atto488-DOPE (DOPE*) and Alexa488-labeled Myo1b (Myo1b*) in a SLB with bound Myo1b, with or without 0.3 % methylcellulose (MEC), and in absence or in the presence of a dense F-actin network (*n* = 30). See Supplementary Fig. 3 for representative FRAP experiments. Bottom: Effective diffusion coefficients of Cherry-CAAX, Cherry-Myo1b, expressed in HEK293T cells (n > 5). Error bars represent s.e.m. **b** Representative kymographs of non-polymerizing (top) and polymerizing F-actin (bottom) in the presence of 0.6 µM G-actin with Myo1b bound to SLBs (Supplementary Movie 8). Scale bar, 5 μm. 1 image/10 s. **c** Dot blot representation of the velocities *v*_*f*_ of stabilized (top) and polymerizing F-actin (bottom) sliding on immobilized Myo1b (dark blue) or on Myo1b bound to a SLB (cyan). The number of analyzed filaments is indicated. **d** Model for filament sliding: The effective filament sliding is determined by a balance between the viscous dissipation of the motor moving with a velocity *v*_*m*_ in the lipid bilayer with a viscosity *η*_*m*_ and a filament sliding at a velocity $\widetilde {v_f}$ in a solution of viscosity *η*_*b*_. **e** Δ*L* versus time for the single filaments shown in (**b**). **f** *v*_*p*_ as a function of G-actin concentration *C*_*m*_ for the different conditions. The fit to the data is the same as in Fig. 2d. Inset: *k*_*off*_ for the different conditions. Error bars represent s.e.m. (*n* > 25). Source data are provided as a Source Data file. **g** Model for force transmission: The effective force experienced by the polymerizing filament $\widetilde {F_{\mathrm{mot}}}$ is diminished by the motion in the lipid bilayer of the motor *v*_*m*_at the barbed end

In cells, Myo1b is bound to the fluid plasma membrane lipid bilayer through the interaction of its PH domain with PI(4,5)P2^13^, and thus it is not immobilized. We mimic experimentally these cellular conditions by analyzing the impact of Myo1b on actin dynamics when bound to a glass-supported lipid bilayer (SLB) composed of 79.5% POPC, 20% L-α-phosphatidylinositol-4,5-bisphosphate (PI(4,5)P2) and 0.5% Rhodamine-PE or Atto488-DOPE (mol/mol) (Fig. 1e and Fig. 3) (“Methods”). We checked using fluorescence recovery after photobleaching (FRAP) that membrane fluidity was preserved in the SLB with bound Myo1b (Fig. 3a and Supplementary Fig. 3). The lipid diffusion coefficient was in agreement with data published on SLBs composed of pure POPC^14^. After recruitment on the SLB, Myo1b diffuses freely in the plane of the membrane (Fig. 3a). We did not observe any difference between experiments with or without methylcellulose in the bulk (Fig. 3a). In addition, the lipids continue to diffuse freely even when Myo1b diffusion is strongly decreased by a dense actin network (Fig. 3a) due to an emerging coupling when a filament bridges multiple motors. The diffusion coefficients are close to those measured in cell membranes (Fig. 3a), showing that in our in vitro experiments, the fluidity of the membrane is preserved. As previously reported^15^, myosin 1 proteins bound to a lipid bilayer exert a force strong enough to propel actin filaments in spite of the fluidity of the support. We confirmed that in the presence of 2 mM ATP and at a similar Myo1b density as when immobilized (8500 µm^−2^), stabilized and polymerizing F-actin slides on Myo1b bound to SLBs, although with a velocity reduced by about 25%: *v*_*f*_  = 37.6 ± 7.3 nm.s^−1^ and *v*_*f*_  = 39.3 ± 8.2 nm s^−1^ respectively (Fig. 3b, c, Supplementary Movie 8 and Supplementary Table 1).

We have calculated the relative contributions of the viscous drag of the bulk and of the lipid bilayer on the motion of the filaments (Supplementary Note). First, we have considered F-actin moving in water ($\eta _b = 10^{ - 3}Pa.s$) above Myo1b bound to a SLB (Fig. 3d). We estimate that, since the in-plane viscous drag between the motor and the lipid bilayer is much larger than the bulk viscosity experienced by the actin filaments, the velocity of the motors bound to actin filaments with respect to the bilayer couple, *v*_*m*_, practically vanishes. Thus, filaments slide with a velocity $\widetilde {v_f}$ similar to that measured for immobilized motors:$\widetilde {v_f} \approx v_f$ (Supplementary Fig. 4). Including the increased viscosity of the bulk in the presence of methylcellulose (10^−2^ Pa s at 0.3%, product information Sigma) and crowding effects between nearby filaments reduces the effective sliding speed of the filament $\widetilde {v_f}$ since part of the sliding is dissipated by in-plane motion of the motors in the bilayer (Supplementary Fig. 4). This can explain why in our experiments, F-actin moves over SLB-bound Myo1b but with a slightly reduced velocity as compared to immobilized Myo1b (Fig. 3c, Supplementary Table 1). This is in line with the results by Grover et al.^16^ showing a decreased gliding velocity of membrane-anchored kinesins due to their slippage in the lipid bilayer.

In these experimental conditions, we observed a significant increase of the actin depolymerization rate at the barbed end *k*_off_ when filaments slide on Myo1b bound to a SLB, although weaker than for immobilized Myo1b, while keeping the polymerization rate unchanged (Fig. 3e, f and Supplementary Table 2). We conclude that the dissipation of sliding filaments in SLBs is low enough to let Myo1b exert a significant dissociation force even when bound to a fluid membrane (See force balance in Fig. 3g).

## Discussion

As previously shown, MyoII induces actin network contraction, potentially leading to filament buckling and breaking^17,18^. However, we show here that muscle MyoII does not affect actin polymerization dynamics. Different actin-binding proteins are already known for preventing actin polymerization (capping protein)^11^, enhancing it (formin)^19,20^ or depolymerizing actin (ADF/cofilin)^21,22^ at the barbed end. Also, some kinesin motors, e.g., kinesins 8 and 13, have been shown to depolymerize microtubules^23,24^. We demonstrate here that Myo1b, but not MyoII, induces a significant actin depolymerization at the barbed end (Supplementary Tables 1 and 2). This suggests that a mechanical process is involved in actin monomers’ removal at the actin filament tip. One possible mechanism could be through the modulation of the torsion of the filaments^25^. In this case, the polymerization kinetics is expected to depend on the filament length with a twist gradient inversely proportional to the length. However, this is not what we observe (Supplementary Fig. 2h), excluding an explicit role of filament torsion due to motor attachment along the filament. Since the effect is essentially detected at the extremity of the filament, a local process should account for depolymerization by Myo1b. Given the motor activity of Myo1b, the increased actin depolymerization could be of mechanical origin and due to a force exerted on the filament by the last Myo1b motor close to its barbed end. Any mechanism where a motor at the barbed end has a longer attachment time would create a force opposing the motion of the actin filament that could lead to an enhanced actin dissociation at this extremity. This force and therefore the depolymerization rate increase with the filament velocity and with the attachment time of the last Myo1b to the filament. We could for instance consider that Myo1b has specific molecular properties when bound at the barbed end, such as a higher affinity or a different type of interaction with this part of the filament. The stronger binding could also have a mechanical origin, such as a catch-bond effect. Myo1b is indeed a strong catch-bond motor in the few pN force range^8^, as compared to MyoII^12^. However, single molecule experiments have evidenced that the motor has an enhanced attachment time when it resists to a load^8^, but this does not correspond to our experimental situation. Very few experiments have been performed with assisting loads^26^, only on a very low force range, not with the full-length Myo1b and far from actin extremity; thus, some unexpected load-induced detachment reduction might occur in our conditions. Experiments that could provide molecular details on the interaction of Myo1b with actin filaments plus-end are obviously extremely challenging, but nevertheless, we have uncovered a peculiar behavior of Myo1b at this filament tip with our gliding assay.

These observations indicating that Myo1b is an actin depolymerase, even when bound to a lipid bilayer, suggest together that this myosin is able to regulate actin dynamics in vivo nearby different cellular membranes. Myo1b’s influence on actin dynamics can control the organization of actin networks, as reported in growth cones^6^. An actin network can be impacted by Myo1b in different ways. It can reduce the length of actin filaments, as shown by this work, and thus change the mesh-size, or the cortical thickness and consequently the cortical contractibility^27^. Whether or not it can affect the Arp2/3-dependent branched actin network and/or formin-dependent actin bundles remains to be explored. Moreover, since Myo1b is specifically present at the interface between the plasma membrane and the cortical actin, Myo1b may coordinate receptor signaling by arranging the cytoskeleton^28^. Further experiments need to be performed in the future to determine the relative contribution of Myo1b with respect to the other proteins that regulate actin dynamics.

Experimental evidence supports a role of several myosin 1 proteins in membrane remodeling^3^. Similarly to capping proteins^29^, Myo1b and perhaps other myosin 1 proteins could shape membranes by regulating the growth of filaments at the plasma membrane. Alternatively, Myo1b could shape membranes by inducing stresses in the cortical actin. Indeed, Myo1b induces actin movement and reduces actin growth when bound to supported bilayers, as shown in our experiments. Since the fluidity of our synthetic membranes and of cellular membranes are similar (Fig. 3a), we propose that Myo1b has the same function in cells. Collectively, these motors could drive the sliding of actin filaments at the membrane surface, which could create stresses that relax by deforming the cortex and the attached membrane. Interestingly, when Myo1b is bound to a deformable giant liposome, we observed that it produces membrane invaginations in presence of stabilized actin filaments (Supplementary Fig. 5).

Besides myosin II and myosin 1 proteins, myosin VI has also been reported to influence the actin architecture during, e.g., spermatid individualization in Drosophila^30^ or around melanosomes^31^. It might be time now to take a fresh look on the involvement of non-conventional myosins in actin dynamics and organization.

## Methods

### Protein purification

Actin was purified from rabbit muscle and isolated in monomeric form in G buffer (5 mM Tris-HCl, pH 7.8, 0.1 mM CaCl_2_, 0.2 mM ATP, 1 mM DTT and 0.01% NaN_3_). Actin was labeled with Alexa 594 succimidyl ester-NHS^32^.

Myosin II was purified from rabbit muscle following the method described by Pollard^33^.

Expression and purification of Myosin 1b: FLAG-myo1b was expressed in HEK293-Flp-In cells (ThermoFischer Scientific)^4^ cultured in Dulbecco’s modified Eagle medium supplemented with 10% fetal bovine serum and 0.18 mg ml^−1^ hygromycine in a spinner flask at 37 °C under 5% CO_2_, and collected by centrifugation (1,000 g, 10 min, 4 °C) to obtain a 4–5 g of cell pellet. The pellet was lysed in FLAG Trap binding buffer (30 mM HEPES, pH 7.5, 100 mM KCl, 1 mM MgCl_2_, 1 mM EGTA, 1 mM ATP, 1 mM DTT, 0.1% protease inhibitor cocktail (PIC), 1% Triton X-100) for 30 min at 4 °C and centrifuged at 3400 × *g* for 10 min at 4 °C. The collected supernatant was then ultracentrifuged (250,000 × *g*, 60 min, 4 °C). The solution between pellet and floating lipid layer was incubated with 150 µl of anti-FLAG beads for 2 h at 4 °C. The beads were collected by centrifugation (1000 × *g*, 5 min, 4 °C). After a washing step, FLAG-myo1b was then eluted by incubating with 0.24 mg ml^−1^ of 3X FLAG peptide in 300 µl elution buffer (binding buffer without Triton X-100 supplemented with 0.1% methylcellulose) for 3 h at 4 °C. After removal of the beads by centrifugation (1000 × *g*, 3 min, 4 °C), the protein solution was dialyzed against elution buffer overnight at 4 °C to remove the 3X FLAG peptide. Myo1b was fluorescently labeled using Alexa Fluor 488 5-SDP ester^34^. Inactivated Myo1b was removed by ultracentrifugation (344,000 × *g*, 20 min, 4 °C) with 10 µM F-actin in presence of 2 mM ATP. Inactivated Myo1b was then dissociated from F-actin by incubating the pellet collected after untracentrifugation in elution buffer (30 mM HEPES, pH 7.5, 100 mM KCl, 1 mM MgCl_2_, 1 mM EGTA, 1 mM ATP, 1 mM DTT and 0.1% methylcellulose) supplemented with 1 M NaCl and collected in the supernatant after a second centrifugation (344,000 × *g*, 20 min, 4 °C).

### Supported lipid bilayer (SLB) preparation

SLBs were formed by fusion of small unilamellar vesicles (SUVs) prepared as follows. Lipid mixtures containing 79.5% POPC, 20% L-α-phosphatidylinositol-4,5-bisphosphate (PI(4,5)P_2_) and 0.5% Rhodamine-PE or Atto488-DOPE (mol/mol) were mixed together in a glass vial, dried with N_2_, placed in vacuum desiccator for 1 h, then rehydrated with Fluo F buffer (5 mM Tris-HCl- pH 7.8, 100 mM KCl, 1 mM MgCl_2_, 0.2 mM EGTA, 0.2 mM or 2 mM ATP, 10 mM DTT, 1 mM DABCO, 0.01% NaN_3_) for 30 min at room temperature, to a final lipid concentration of 2 mg/mL. After rehydration, the glass vial was vortexed to detach the liposomes. SUVs were formed by sonication, aliquoted and stored at −20 °C. For SLB formation by fusion, CaCl_2_ was added to a final concentration of 5 mM, with 50 µl of SUVs. The solution was incubated in the chamber for 20 min and washed five times with Fluo F buffer 0.1% BSA. The quality of the SLB was checked by FRAP.

### Giant unilamellar vesicle (GUV) preparation

Lipid compositions for GUVs were 79.7% POPC, 20% L-α-phosphatidylinositol-4,5-bisphosphate (PI(4,5)P_2_) and 0.3% Texas Red DHPE. GUVs were prepared by using polyvinyl alcohol (PVA) gel-assisted method in a 200 mM sucrose buffer at room temperature for 2 h as described previously^35^.

### Myosin 1b surface density

We measured the protein surface density (number of proteins per unit area) on solid surfaces or on SLBs by using a previously established procedure^36,37^. It is calculated from a labeled proteins/lipids calibration. We first measure the fluorescence of POPC SLBs containing predefined amounts of Atto488-DOPE fluorescent lipids (DOPE*) to establish the relationship between the density of DOPE* $n_{\mathrm{DOPE} ^\ast }$ and the corresponding fluorescence intensity $I_{\mathrm{DOPE} ^\ast }^{\mathrm{SLB}}$ (Supplementary Fig. 1b). Assuming an area per POPC of 0.68 nm^2^, we derive the calibration coefficient A corresponding to the slope of this curve. Note that A depends on the illumination and recording settings of the microscope.

1 $$n_{\mathrm{DOPE} ^\ast = A \times I_{\mathrm{DOPE} \ast }^{\mathrm{SLB}}}$$

Since Myo1b is labeled with Alexa488 and not Atto488, we have to correct this value by the ratio of fluorescence of the two fluorescent dyes in bulk deduced from the slope of the titration curves $\frac{{I_{\mathrm{Alexa}488}}}{{I_{\mathrm{DOPE}^ \ast }}}$ (Supplementary Fig. 1c, d). We then obtained the surface density of the protein deduced from the measurement of the Myo1b-Alexa488 intensity $I_{\mathrm{Myo1b} ^\ast }$ as:

2 $$n_{\mathrm{Myo1b}} = \frac{A}{{\frac{{I_{\mathrm{Alexa}488}}}{{I_{\mathrm{DOPE}^ \ast }}} \times Z}} \times I_{\mathrm{Myo1b} ^\ast }$$

where *Z* is the degree of labeling for the protein of interest (Here, *Z*=1). In our experiments, the calibration factor $\frac{A}{{\frac{{I_{\mathrm{Alexa}488}}}{{I_{\mathrm{DOPE}^ \ast }}} \times Z}}$ is equal to 0.278.

### Single-filament TIRF microscopy assays

The kinetics of single filament assembly was monitored by TIRF microscopy (Eclipse Ti inverted microscope, 100× TIRF objectives, Quantem 512SC camera). The experiments were controlled using the Metamorph software. Coverslips and glass slides were sequentially cleaned by sonication with H_2_O, ethanol, acetone for 10 min, then 1 M KOH for 20 min and H_2_O for 10 min. In the case of SLB, first the coverslips and glass slides were cleaned by sonication with Hellmanex III (Hellma Analytics) for 30 min. Flow chambers were assembled with a coverslip bound to a glass slide with two parallel double-stick tapes. The chamber was incubated with 100 nM anti-myo1b antibody in G buffer (5 mM Tris-HCl, pH 7.8, 0.1 mM CaCl_2_, 0.2 mM ATP, 1 mM DTT and 0.01% NaN_3_) for 10 min at room temperature. The chamber was rinsed three times with buffer G 0.1 % BSA and incubated 5 min at room temperature. Then the chamber was incubated with 300 nM Alexa488-labeled myo1b in Fluo F buffer (5 mM Tris-HCl, pH 7.8, 100 mM KCl, 1 mM MgCl_2_, 0.2 mM EGTA, 0.2 mM or 2 mM ATP, 10 mM DTT, 1 mM DABCO, 0.01% NaN_3_) for 10 min at room temperature. Assays were performed in Fluo F buffer, containing 0.2 or 2 mM constant ATP, supplemented with 0.3% methylcellulose (Sigma) and with G-actin (10% Alexa594) or F-actin (stabilized with phalloidin-Alexa594) at indicated concentrations. To maintaining a constant concentration of ATP in this assay an ATP regenerating mix, including 2 mM ATP, 2 mM MgCl_2_, 10 mM creatine phosphate and 3.5 U/mL creatine phosphokinase, which constantly re-phosphorylates ADP into ATP to maintain a constant concentration of free ATP, was added.

The sliding and elongation velocities of actin filaments were analyzed by using Kymo Tool Box plugin of Image J software (https://github.com/fabricecordelieres/IJ_KymoToolBox). Only filaments longer than 20 pixels are analyzed. When filaments slide on myosins, only those moving directionally during the whole sequence are selected. On each image of a sequence, a segmented line is manually drawn over a single filament, which generates a 10 pixel wide band. The plugin flattens the curved filaments and generates a kymograph. The accuracy on the displacement and the length of the filaments is of the order of the pixel size (160 nm). We consider that each actin subunit contributes to 2.7 nm of the filament length.

### FRAP methods

For diffusion measurements, FRAP experiments were performed through a ×100 or ×60 oil immersion objective on an inverted spinning disk confocal microscope (Nikon eclipse Ti-E equipped with a Prime 95B™ Scientific CMOS camera, Photometrics) equipped with a FRAP unit. Recovery curves (average of five independent experiments, performed on different circular regions of the SLB using the same bleaching conditions) were normalized to the initial intensity and fitted with a single exponential function. We derive the *τ*_1/2_ time corresponding to the time at which the fluorescence signal has recovered 50% of its value before bleach. We calculated the diffusion coefficient using the Soumpasis equation^38^:

3 $$D_r = 0.224\frac{{r^2}}{{\tau _{1/2}}}$$

where *r* is the radius of the bleached region.

### Reporting summary

Further information on research design is available in the Nature Research Reporting Summary linked to this article.

## Supplementary information

Supplementary Information

Supplementary Movie 1

Supplementary Movie 2

Supplementary Movie 3

Supplementary Movie 4

Supplementary Movie 5

Supplementary Movie 6

Supplementary Movie 7

Supplementary Movie 8

Reporting Summary

Description of Additional Supplementary Files

Source Data File

## Data Availability

Data supporting the findings of this manuscript are available from the corresponding authors upon reasonable request. A reporting summary for this Article is available as a Supplementary Information file. The source data underlying Figs. 2d, 3f are provided as a Source Data file.
