# Supplementary material for: Myosin 1b is an actin depolymerase
Source: Nat Commun. 2019 Nov 15;10:5200. doi: 10.1038/s41467-019-13160-y (PMC6858320; doi:10.1038/s41467-019-13160-y)
Supplement: Supplementary file 1 — Supplementary Information [file 41467_2019_13160_MOESM1_ESM.pdf]

Supplementary Information

Myosin 1b is an actin depolymerase

Pernier et al.

Supplementary Note

Filament sliding on a lipid bilayer

In order to estimate how the motor force is transmitted to the filament when the molecular motors are immersed in a lipid bilayer, instead of being rigidly anchored to a solid surface, we write a simplied force balance between the viscous friction force of the motor/filament and the force exerted by the molecular motors nF_{mot} where $n = \rho l$ is the number of attached motors along the filament of length l ,

$$-\xi_f \widetilde{v_f} = nF_{mot} \quad \text{(E1)}$$

$$-\xi_f \widetilde{v_f} = -n\xi_m v_m \quad \text{(E2)}$$

where ξ_m is the in-plane friction coeffcient of the motor complex in the lipid bilayer, v_m , the speed of a molecular motor, ξ_f, the friction coefficient between the filament and the surrounding solution and $\widetilde{v_f}$, the speed of the filament in the assay. The first equation is the force balance on the filament and the second equation is the force balance on the filament and motors complex. We use here a simplified expression for the motor force [1],

$$F_{mot} = f_s\,(1 - \frac{v_m - \widetilde{v_f}}{v_0}), \quad \text{(E3)}$$

where f_s is the stall force of one motor and v_0 the motor speed at vanishing external force ($f_s \approx O\,(1)$ pN and $v_0 \approx 50\,nm/s$). The friction between the filament and the solution can be estimated as $\xi_f \approx 2\Pi n_b/\,(\log l/b_f) \approx O\,(10^{-8})Pa.s.m$, where we use the bulk viscosity of water $n_b = O\,(10^{-3})Pa.s$ and as a cut-off lengthscale, the size of the filament $l = O\,(10^{-5})m$. Note however that the effective bulk viscosity can be significantly larger since the filament slides close to a surface. The friction between the motor complex and the lipid membrane is $\xi_m \approx 4\Pi n_m/\,(\log l_0/L) \approx O\,(10^{-9})Pa.s.m$ [2], where L is the size of the membrane and l_0 the size of a motor (we estimate the membrane viscosity as $n_m \approx O\,(10^{-10})Pa.s$ [2] and $(\log l_0/L) = O\,(1)$). Solving equations SE 1 and SE 2 gives the following values for the velocity of the filament, $\widetilde{v_f}$, relative to the velocity at zero external force on a solid substrate, v_0,

$$\frac{\widetilde{v_f}}{v_0} = -\frac{n\xi_m}{\xi_f + n\xi_m + v_0\xi_f\xi_m/f_s}, \quad \text{(E4)}$$

and for the velocity of the motor, v_m ,

$$\frac{v_m}{v_0} = -\frac{\xi_f}{\xi_f + n\xi_m + v_0\xi_f\xi_m/f_s}, \quad \text{(E5)}$$

For realistic values of the friction coefficient of water and typical force values we obtain a filament speed which is very close to the filament speed on a solid substrate $\frac{\widetilde{v_f}}{v_0} \approx 1$, indicating that, since the in-plane membrane friction of the motor is larger than the filament friction with the fluid, the motors are effectively immobile. However, upon increasing the viscous friction between the filament and the bulk by one/two orders of magnitude, potentially due to inter-filament friction (at high filament density) or to the addition of methylcellulose, the sliding speed of the filament diminishes significantly (Supplementary figure 4). Also decreasing the density of motors along the filament impacts the sliding speed since the effective friction between membrane and motor is proportional to the density of motors.

Supplementary Tables

Table S1

	Myosin	[ATP] (mM)	Density (molecules/µm^2)	Condition	Velocity (nm/s)
On glass	Myosin 1b	2	8393 ± 430	No polymerization	56.4 ± 15.4
		2	8444 ± 873	Polymerization	53.9 ± 5.5
		0.2	8776 ± 1293	No polymerization	24.7 ± 4.2
		0.2	8876 ± 459	Polymerization	27.2 ± 1.9
		2	447 ± 20	No polymerization	33.3 ± 3.1
		2	533 ± 84	Polymerization	35.6 ± 6.2
	Myosin II	2	Not reported	No polymerization	257 ± 76
		2	Not reported	Polymerization	279 ± 68
On SLB	Myosin 1b	2	8770 ± 170	No polymerization	37.6 ± 7.3
		2	8657 ± 1251	Polymerization	39.3 ± 8.2

Supplementary Table 1: Sliding velocities v_f of stabilized and polymerizing actin filaments on Myo1b or Myosin II in the different used conditions.

Table S2

	Myosin	[ATP] (mM)	Density (molecules/µm^2)	k_{on} (su.µM^{-1}.s^{-1})	k_{off} (su.s^{-1})	Critical concentration C_{c+} (µM)
In solution	No Myosin	0.2		10.3 ± 0.8	1.1 ± 0.4	0.10 ± 0.05
	No Myosin	2		10.9 ± 0.6	1.4 ± 0.4	0.13 ± 0.06
	Myosin 1b	2	Not reported	11.4 ± 1.1	1.8 ± 0.5	0.16 ± 0.04
On glass	Myosin 1b	2	8444 ± 873	10.3 ± 0.5	3.2 ± 0.4	0.31 ± 0.05
		0.2	8876 ± 459	10.7 ± 0.9	2.1 ± 0.5	0.20 ± 0.04
		2	533 ± 84	10.0 ± 1.2	2.6 ± 0.6	0.26 ± 0.08
	No motor	2	7767 ± 423	10.9 ± 0.8	1.6 ± 0.5	0.15 ± 0.05
	Myosin II	2	Not reported	10.0 ± 0.8	1.0 ± 0.5	0.10 ± 0.05
On SLB	Myosin 1b	2	8657 ± 1251	9.9 ± 0.8	2.5 ± 0.5	0.26 ± 0.05

Supplementary Table 2: Rate constants of G-actin monomer association and dissociation in the absence and presence of Myo1b or Myosin II in the different used conditions.

Supplementary Figures

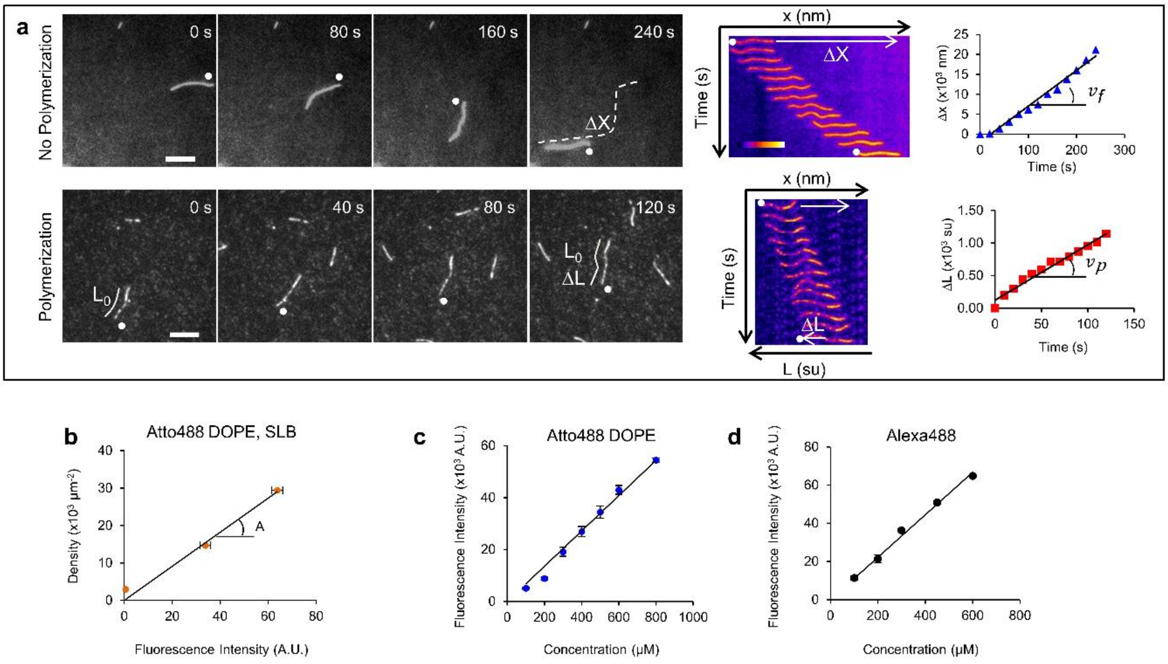

Supplementary Figure 1: Analysis of the experimental data and calibration of the Myo1b density. (a) Left: Time-lapse images obtained by TIRF microscopy of a stabilized filament (top) (and a polymerizing filament in the presence of 1.2 μM G-actin (bottom) sliding along glass-anchored Myo1b (see movie 1). White dots indicate the filament's barbed end. The white dashed represents the trajectory of the stabilized filament, and *ΔX* the total displacement of the filament over the period considered. L_0 and *ΔL* are the initial length of the polymerizing filament and its elongation, respectively, both normalized by the actin sub-unit length. Middle: corresponding kymographs. The sliding *ΔX* and the elongation *ΔL* correspond to the white arrows. Right: Time variation of *ΔX* and *ΔL*. The sliding velocity v_f and the elongation rate v_p are deduced from the slopes of the graphs. Actin fluorescence intensity is represented according to the "Fire" LUT of Image J. Scale bar, 5μm. 1 image/20 sec. **(b-d)** Myo1b density on the solid substrate or on the supported bilayer deduced from (b) the measurement of the fluorescence intensity of a reference lipid Atto488DOPE at known density in a SLB (supported lipid bilayer), and the comparison of (c) the fluorescence of Myo1b dye Alexa 488 and (d) Atto488DOPE in bulk at known concentrations (see Methods). The calibration constant *A* is deduced from the slope of b).

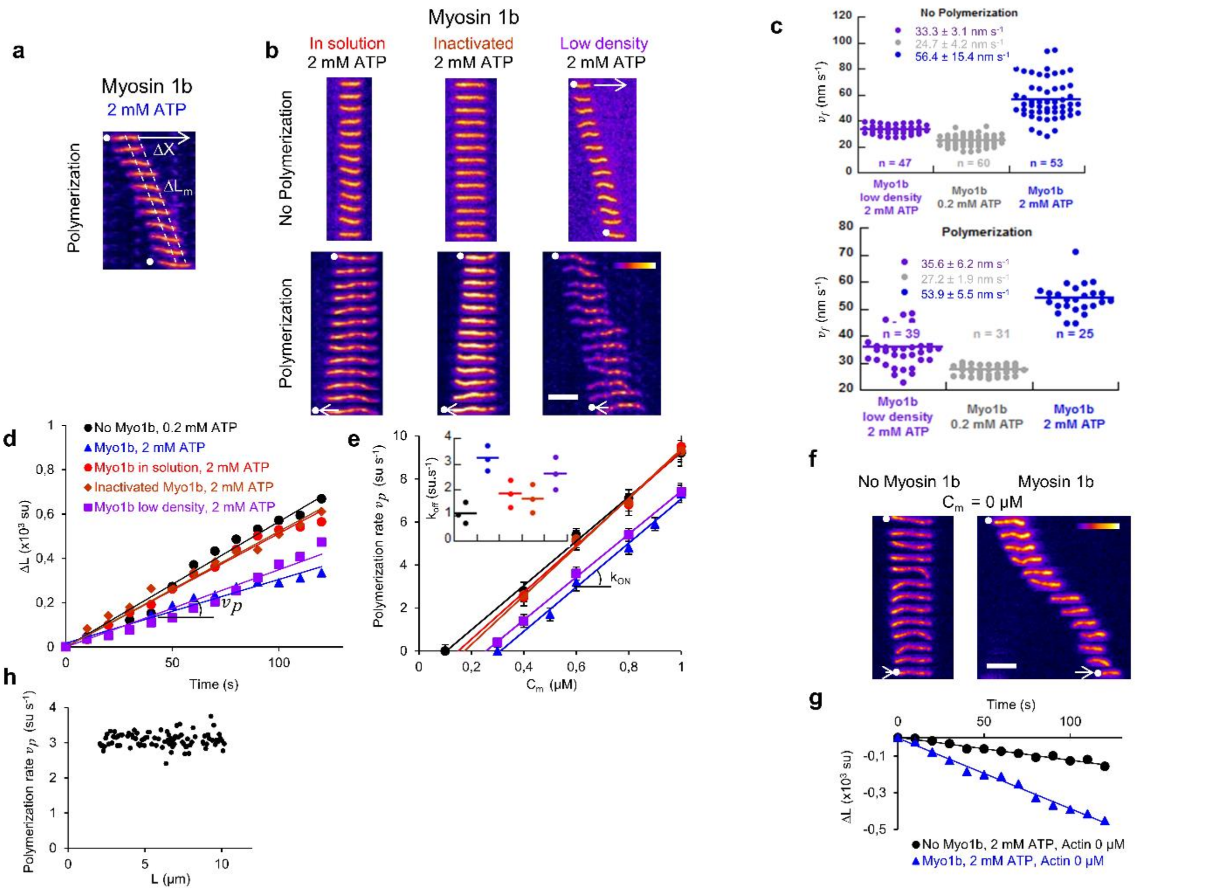

Supplementary Figure 2: Impact of Myo1b on F-actin pointed end and impact of Myo1b at low density, inactivated, or in solution on the sliding and actin depolymerization at the barbed end. **(a)** Representative kymograph of polymerizing actin filaments, in presence of 0.6 µM G-actin, 2 mM ATP with anchored Myo1b at ≈ 8000 µm^{-2} (see movie 4). The elongation ΔL_m of the filaments at the pointed end (between the 2 dashed white lines) is indicated. Scale bar, 5 µm. 1 image/10 sec. **(b)** Representative kymographs of phalloidin stabilized filaments (top) or polymerizing actin filaments (bottom), in presence of 0.6 µM G-actin, 2 mM ATP, with Myo1b in solution (red), Myo1b without motor activity (brown), with anchored Myo1b at low density (≈500 motor/µm^2) (purple). (See movies 2, 3 and 5). Scale bar, 5µm. 1image/10 sec. **(c)** Comparison of the distribution of the velocities v_f of stabilized (top) and polymerizing F-actin (bottom) sliding on immobilized high density Myo1b, 2 mM ATP (dark blue) and 0.2 mM ATP (grey) and low density Myo1b, 2 mM ATP (purple). Velocity distributions and average velocities are indicated. Data are represented with a "Dot plot". The number of analyzed filaments is indicated. **(d)** ΔL versus time for the single filaments for the conditions shown in (a) and (b) and in the absence of Myo1b. **(e)** v_p as a function of G-actin concentration C_m for the different indicated conditions. The fit to the data is the same as in Fig. 2d. Error bars represent s.e.m. (n > 25). Inset: k_{off} for the different conditions. **(f)** Representative kymographs of depolymerizing actin filaments, in absence of G-actin (C_m= 0 µM), without Myo1b (left) or with anchored Myo1b at ≈8000 motor/µm^2 (right), with 2 mM ATP. (See movie 7). Scale bar, 5µm. 1 image/10 sec. **(g)** ΔL versus time for the single filaments shown in (f). **(h)** v_p as a function of filament length L for single polymerizing filaments sliding along glass-anchored Myo1b in the presence of 0.6 µM G-actin and 2 mM ATP (n = 90, v_p measured during 30 sec).

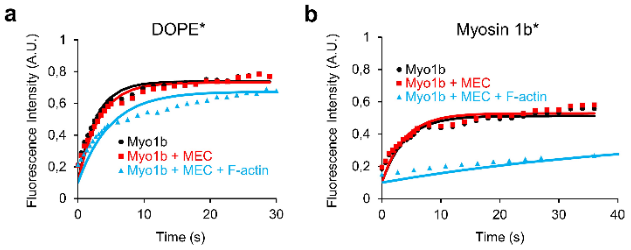

Supplementary Figure 3: FRAP data of DOPE and Myo1b in a SLB. (a and b) Representative FRAP recovery curves (symbols) and the best fit with single exponential (solid line) of Atto488-DOPE (DOPE*) and Alexa488-labelled Myo1b (Myosin 1b*) in a SLB with bound Myo1b, with (in red) or without (in black) 0.3 % methylcellulose (MEC), and in absence or presence (in cyan) of a dense F-actin network.

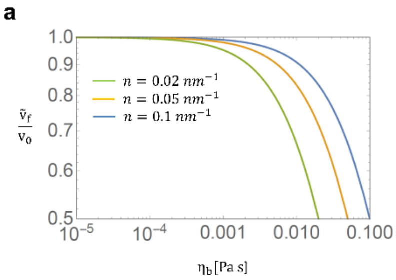

Supplementary Figure 4: Effect of bulk viscosity on relative velocity of the filament. Velocity of the filament $\widetilde{v_f}$, relative to its velocity on a solid substrate v_0 (Supplementary note Eq. E4), for different values of motor density n. Increasing the bulk viscosity, relative to the membrane viscosity, induces motion of the motors in the bilayer, hence decreasing the effective velocity of the filament. Increasing the density of molecular motors on the surface increases the effective membrane friction and hence increases the sliding speed.

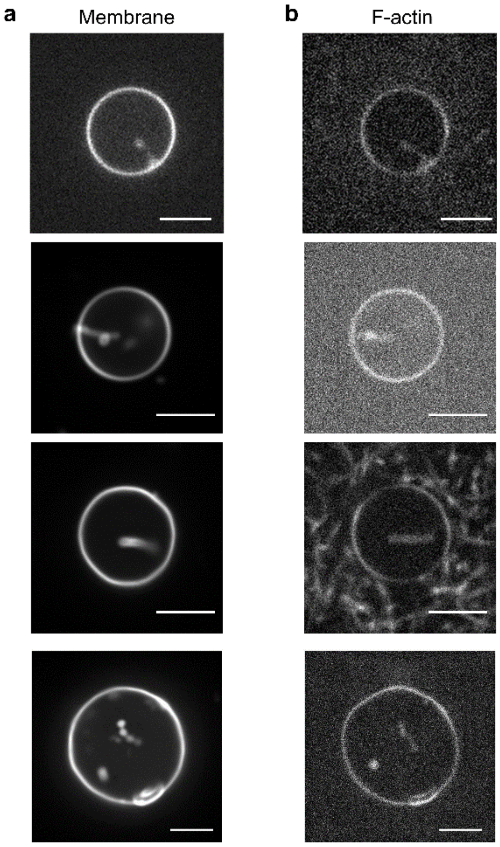

Supplementary Figure 5: Myo1b bound to giant liposomes produces membrane invagination in presence of stabilized actin filaments. Representative confocal microscopy images of tubules induced by Myo1b bound to a PI(4,5)P_2-containing GUV in the presence of stabilized actin filaments. We have observed tubulation in the equatorial plane for 16 GUVs over a total of 96. Labeling corresponds to **(a)** 0.3% Texas Red DHPE (mol/mol) and **(b)** stabilized actin filaments with Alexa Fluor 647 phalloidin. Scale bars, 5 µm.

Supplementary references

1. Vilfan, A., Frey, E. & Schwabl, F. Force-velocity relations of a two-state crossbridge model for molecular motors. *Europhys. Lett.* **45**, 283-289 (1999).

2. Saffman, P. G. & Delbrück, M. Brownian motion in biological membranes. *Proc. Natl Acad. Sci. USA* **72**, 3111-3113 (1975).
