# Supplementary material for: Myosin 1b is an actin depolymerase
Source: Nat Commun. 2019 Nov 15;10:5200. doi: 10.1038/s41467-019-13160-y (PMC6858320; doi:10.1038/s41467-019-13160-y)
Supplement: Supplementary file 11 — Description of Additional Supplementary Files [file 41467_2019_13160_MOESM11_ESM.pdf]

Title: Supplementary Movie 1: Stabilized and polymerizing actin filaments sliding along glass-anchored Myo1b (corresponding to Supplementary Figure 1a).

Description: Sliding of stabilized filaments labeled with phalloidin-Alexa547 (a) and polymerizing filaments (b) with 1.2 µM actin in bulk (10 % Alexa594 labeled), along Myo1b (8000 μm^{-2}) at 2 mM ATP. White arrow indicates the initial position of the filament corresponding to the kymograph in Fig. S1a. Note that no filament breaking is observed during these experiments. Scale bar 5 µm. Time in s.

Title: Supplementary Movie 2: Effect of the ATP concentration and Myo1b density on stabilized actin filaments sliding along glass-anchored Myo1b (corresponding to Figs. 2a and Supplementary Figure 2b).

Description: Filaments stabilized with phalloidin-Alexa547 as a function of ATP concentration (0.2 and 2 mM ATP), without Myo1b (a), sliding on Myo1b at high density (8000 μm^{-2}) (b, c) or at low density (400 μm^{-2}) (d). Wide field movies followed by single filament movies corresponding to kymographs shown in Fig. 2a-Top panel and Fig. S2b-Top panel. White arrows indicate the initial position of single filaments. Scale bar 5 µm. Time in s.

Title: Supplementary Movie 3: Effect of ATP concentration and Myo1b density on polymerizing actin filaments sliding along glass-anchored Myo1b (corresponding to Supplementary Figure 2a).

Description: Polymerizing filaments as a function of ATP concentration as a function of ATP concentration (0.2 and 2 mM ATP), without Myo1b (a), sliding on Myo1b at high density (8000 μm^{-2}) (b, c) or at low density (400 μm^{-2}) (d). Wide field movies followed by single filament movies corresponding to kymographs shown in Fig. 2a-Bottom panel and Fig. S2b-Bottom panel. White arrows indicate the initial position of single filaments. Scale bar 5 µm. Time in s.

Title: Supplementary Movie 4: No effect at pointed-end on polymerizing actin filaments sliding along glass-anchored Myo1b.

Description: Polymerizing actin filaments with 0.6 µM actin (10 % Alexa594 labeled), along Myo1b (8000 μm^{-2}) at 2 mM ATP. The white arrow indicates the initial position of the filament shown in Fig. S2a. Scale bar 5 µm. Time in s.

Title: Supplementary Movie 5: Stabilized and polymerizing actin filaments with Myo1b in bulk or inactivated (corresponding to Supplementary Figure 2b).

Description: Filaments stabilized with phalloidin-Alexa547 and polymerizing actin filament, with 0.6 µM actin (10 % Alexa594 labeled), 2 mM ATP, 300 nM Myo1b in the bulk, or bound but inactivated. Note that in the bottom left movie, one filament seems to move but it suddenly appears in the field of view while sedimenting. Frames correspond to the kymographs shown in Fig. S2b; arrows indicate the initial position of these filaments. Scale bar 5 µm. Time in s.

Title: Supplementary Movie 6: Stabilized and polymerizing actin filaments sliding along glass-anchored MyoII (corresponding to Fig. 2a).

Description: Filaments stabilized with phalloidin-Alexa547 or polymerizing at 0.6 µM actin (10 % Alexa594 labeled), sliding along MyoII at 2 mM ATP. Frames correspond to the kymographs shown in Fig. 2a; arrows indicate the initial position of these filaments. Scale bar 5 µm. Time in s.

Title: Supplementary Movie 7: Impact of Myo1b on the depolymerization of actin filaments in the absence of G-actin in the bulk (corresponding to Supplementary Figure 2f).
Description: Filaments depolymerizing in the absence of G-actin in bulk, without Myo1b, or sliding along Myo1b, at 2 mM ATP. Frames correspond to the kymographs shown in Fig. S2f; arrows indicate the initial position of these filaments. Scale bar 5 µm. Time in s.

Title: Supplementary Movie 8: Stabilized and polymerizing actin filaments sliding along Myo1b bound to SLBs (corresponding to Fig. 3b).
Description: Sliding of filaments stabilized with phalloidin-Alexa547 or polymerizing at 0.6 µM actin (10 % Alexa594 labeled), along Myo1b bound to SLBs (≈ 8500 μm^{-2}) at 2 mM ATP. Wide field movies followed by single filament movies corresponding to kymographs shown in Fig. 3b. White arrows indicate the initial position of single filaments. Scale bar 5 µm. Time in s.
